# ﻿*Ophiostoma babimostense* and *Sporothrix europaea* (Ascomycota, Ophiostomatales), two new ophiostomatalean species, associated with ambrosia and bark beetles in Norway and Poland

**DOI:** 10.3897/mycokeys.123.155588

**Published:** 2025-10-13

**Authors:** Robert Jankowiak, Halvor Solheim, Piotr Bilański, Filip Kawa

**Affiliations:** 1 Department of Forest Ecosystems Protection, University of Agriculture in Krakow, Al. 29 Listopada 46, 31-425 Krakow, Poland University of Agriculture in Krakow Krakow Poland; 2 Norwegian Institute of Bioeconomy Research, P.O. Box 115, 1431 Ås, Norway Norwegian Institute of Bioeconomy Research Ås Norway

**Keywords:** Beetle-associated fungi, hardwood, phylogenetics, *Pinus sylvestris*, taxonomy, two new taxa

## Abstract

The order Ophiostomatales includes many species important for forestry, causing plant diseases. They are common associates of bark- and wood-dwelling beetles. Two new ophiostomatalean fungi viz. *Ophiostoma babimostense***sp. nov.** and *Sporothrix europaea***sp. nov.** are proposed, based on morphological characters and multigene phylogenies. *Ophiostoma babimostense* belongs to the *Ophiostoma ulmi* species complex and was isolated from fallen shoots of Scots pine pruned by *Tomicus* species in Poland. The fungus is characterised by the production of a typical pesotum-like and sporothrix-like asexual morphs. *Sporothrix europaea* belongs to the *Sporothrix gossypina* complex and was isolated from hardwood-infested by ambrosia and bark beetles in Poland and Norway. It is characterised by the occurrence of both a sexual and asexual morphs, with long necked ascomata bearing ostiolar hyphae and a sporothrix-like asexual morph.

## ﻿Introduction

Ophiostomatalean fungi (Ophiostomatales, Ascomycota) are characterised by the formation of flask-shaped perithecia and various asexual morphs, including mononematous or synnematous forms (Wingfield et al. 1993; De Beer and Wingfield 2013). These fungi include tree- or wood-infecting species causing a dark bluish discoloration in the sapwood or serious tree diseases and some of them are also the causal agents of human diseases. Ophiostomatalean species are well known to be closely associated with bark- and wood-dwelling beetles and mites (De Beer et al. 2022).

In Europe, surveys for ophiostomatalean fungi from different habitats are constantly improving. Combined with multi-locus molecular phylogenetic analysis has led to the identification of many species of ophiostomatalean fungi, including dozens of novel species (e.g. Linnakoski et al. (2016); Jankowiak et al. (2017, 2019b)). In particular, extensive surveys have been conducted on the diversity and taxonomy of ophiostomatalean and microascalean fungi in Central and Northern Europe. For example, surveys of insect-associated mycobiomes in Norway and Poland, yielded descriptions of almost 30 new species of *Ceratocystiopsis* (Jankowiak et al. 2022), *Graphilbum* (Jankowiak et al. 2020), *Graphium* (Jankowiak et al. 2023a), *Ophiostoma* (Aas et al. 2018; Jankowiak et al. 2019a) and *Sporothrix* (Ostafińska et al. 2021). More recent discoveries generated descriptions of a new genus *Hausneria* (Crous et al. 2024) from a gallery of *Dryocoetes alni* in Norway and the new species, *Ophiostoma juglandis* from *Dryocoetes himalayensis* on *Juglans regia* in Czechia (Májek et al. 2025). In Poland, the screening of ophiostomatalean fungi was conducted from tree wounds (Jankowiak et al. 2019c), in forest soils (Bilański et al. 2023) and in bird’s nests (Błońska et al. 2021), yielding two new soil-inhabiting *Sporothrix* species (Bilański et al. 2023), one new species of *Hawksworthiomyces* from nests of *Ciconia ciconia* (Crous et al. 2023) and three new species of *Leptographium* from tree wounds (Jankowiak et al. 2018a). These surveys revealed a plethora of new beetle-fungus associations and led to the discovery and description of many new species, showing there is still a lot of unknown taxonomic and ecological diversity to be uncovered for the ophiostomatalean fungi in Europe.

*Ophiostoma* is one of the largest genera within the Ophiostomatales and includes tree- or wood-infecting fungi. Currently, more than 170 species are recognised worldwide (De Beer et al. 2022; Bhunjun et al. 2024; Feau et al. 2024; Májek et al. 2025; Wang et al. 2024). Members of *Ophiostoma* are characterised by pigmented ascomata with slender necks and allantoid, reniform, cylindrical to ossiform in side view ascospores. In addition, Hyalorhinocladiella-, Leptographium-, Pesotum- or Sporothrix-like asexual morphs are produced in culture and nature (De Beer et al. 2022). *Ophiostoma* is mostly structured into six phylogenetically well-supported lineages or so-called species complex. They are the *O. clavatum*–, *O. ips*–, *O. minus*–, *O. piceae*–, *O. pluriannulatum*– and *O. ulmi* lineages and species complexes. In addition, some species do not form part of these lineages, such as *O. piliferum* and *O. tetropii* (De Beer et al. 2022; Wang et al. 2024). *Ophiostoma* can be found in a wide range of habitats, but most species are commonly associated with forest trees on which they form more or less stable associations together with bark- and wood-dwelling beetles, mites (Six 2012) and, in a few cases, nematodes (Bhunjun et al. 2024). Some members of *Ophiostoma*, such as *O. piliferum* and *O. minus*, cause the economically important blue-stain in freshly exposed sapwood of softwood species (e.g. Seifert (1993); Uzunović and Byrne (2013); Jankowiak et al. (2021)) or sawn timber (Jankowiak et al. 2018b). In addition, *O. novo-ulmi* is an example of a highly virulent pathogen that has been responsible for Dutch elm disease in Europe, western Asia and North America (Brasier 1991). *Ophiostoma* has a worldwide distribution, but it is especially abundant in the north temperate to boreal conifer ecosystems, in Asia, Europe and North America. They have been observed from *Abies*, *Larix*, *Picea*, *Pinus*, *Pseudotsuga* and *Tsuga*. *Ophiostoma* species also grow from hardwood, such as *Quercus* or *Fagus* (Jankowiak et al. 2019b, c). Some species, such as *Ophiostoma ips*, *O. piceae*, *O. piliferum* and *O. quercus*, are globally widespread, possibly due to human activity and the movement of wood products around the globe (Taerum et al. 2018; Bhunjun et al. 2024).

*Sporothrix* is also a large genus in the Ophiostomatales, with currently 70 recognised species arranged in five phylogenetically well-supported lineages, forming species complex, including the *S. candida*–, *S. inflata*–, *S. stenoceras*–, *S. gossypina*– and *S. pallida* species complexes (De Beer et al. 2016, 2022; Wang et al. 2019; Bilański et al. 2023). Other species form part of several unresolved smaller phylogenetic lineages (Groups D-G) or two lineages *Sporothrix insertae sedis* (XVI & XIX) defined by De Beer et al. (2022). The *S. gossypina* complex is considered as the largest at present, with 18 accepted species. Members of this complex are characterised by the presence of globose ascomatal base with black necks and allantoid to reniform in side view ascospores. The asexual morphs include simple, micronematous to mononematous conidiophores, with denticulate or not denticulate conidiogenous cells showing sympodial growth. Conidia hyaline to that vary in size and shape, mostly subglobose to oblong, obovoid, clavate to strongly curved, guttuliform to fusiform (De Beer et al. 2022). Similar to *Ophiostoma*, members of *Sporothrix* are widely distributed across various climatic zones of the world, colonising diverse environments (De Beer and Wingfield 2013; De Beer et al. 2016). The greatest numbers of species are found on the bark and wood of different forest trees and in the infructescences of *Protea* spp. (e.g. Roets et al. (2013); De Errasti et al. (2016); Bilański et al. (2023)). Other species have been described from soil, ambrosia and bark beetles, mites and from the fruiting bodies of basidiomycetes (e.g. Constantinescu and Ryman (1989); Marmolejo and Butin (1990); De Meyer et al. (2008)). Several species are also well-known as human and animal pathogens (e.g. Lòpez-Romero et al. (2011); Zhang et al. (2015)).

During a survey of ophiostomatalean fungi on hardwoods in Poland and Norway (Aas et al. 2018; Jankowiak et al. 2019b), isolates of an undescribed *Sporothrix* species with a sexual state resembling species in the *S. gossypina* species complex were isolated from different ambrosia and bark beetle species. In addition, isolates of an unknown fungal species with morphological attributes (synnemata) matching those described for species of the *Ophiostoma* were isolated from Scots pine (*Pinus sylvestris* L.) shoots infested by an unknown species of *Tomicus*. The purpose of this study is to characterise these fungi using the morphology, phylogenetic analyses and to formally describe them as novel species of *Sporothrix* and *Ophiostoma*.

## ﻿Materials and methods

### ﻿Isolates and herbarium specimens

A collection of ten Polish and three Norwegian isolates were used in this study. Isolates of *Ophiostoma* sp. were collected in pure Scots pine stands in western Poland (Babimost: 52°09′11″N, 15°50′33″E) in October 2023. Isolations were made from fallen shoots of Scots pine pruned by *Tomicus* spp. as described by Jankowiak and Kolařík (2011). Isolates of *Sporothrix* sp. were collected during surveys of hardwood-infesting bark and ambrosia beetles in Poland and Norway (Aas et al. 2018; Jankowiak et al. 2019b). The cultures are maintained in the culture collection of the Department of Forest Ecosystems Protection, University of Agriculture in Krakow, Poland and in the culture collection of Norwegian Institute of Bioeconomy. The ex-type isolates and representative isolates of the new species described were deposited in the culture collection (CBS) of the Westerdijk Fungal Biodiversity Institute, Utrecht, The Netherlands and in the culture collection (CMW) of the Forestry and Agricultural Biotechnology Institute (FABI), University of Pretoria, Pretoria, South Africa. Dried cultures were deposited as holotype specimens in the National Biodiversity Collection – Herbarium KRAM, the W. Szafer Institute of Botany Polish Academy of Science, Kraków, Poland. Two reference strains were also obtained from collections of CBS. These included a living culture of *Sporothrix fusiformis* (CBS 112912) and *Sporothrix lunata* (CBS 119444) (Table 1).

**Table 1.** Isolates from this study used in the phylogenetic analyses.

Taxa	Previously labelled	Isolate no^A^.			Source	Site	GenBank accessions^B^				
		CBS	CMW	KFL/NIBIO	Host/vector		ITS	LSU	*TUB2*	*TEF1*	*CAL*
*Ophiostoma babimostense* sp. nov.				KFL726J	*Pinus sylvestris*/*Tomicus* sp.	Babimost, Poland	PV350321	PV350321	PV330683	PV330691	PV330700
		CBS 152111 ^C^		KFL728J	*Pinus sylvestris*/*Tomicus* sp.	Babimost, Poland	PV350322	PV350322	PV330684	PV330692	PV330701
				KFL746J	*Pinus sylvestris*/*Tomicus* sp.	Babimost, Poland	PV350323	PV350323	PV330685	PV330693	PV330702
		CBS 152112 ^T^		KFL828J	*Pinus sylvestris*/*Tomicus* sp.	Babimost, Poland	PV350324	PV350324	PV330686	PV330694	PV330703
				KFL836J	*Pinus sylvestris*/*Tomicus* sp.	Babimost, Poland	PV350325	PV350325	PV330687	PV330695	PV330704
*Sporothrix europaea* sp. nov.	*Sporothrix* sp. 4	CBS 151675		NIBIO2016-1656/3/1	*Quercus robur*/*Anisandrus dispar*	Ås, Norway	PV350326	PV350326	PV330688	PV330696	PV330705
		CBS 151676 ^T^		NIBIO2016-1657/2/1	*Quercus robur*/*Anisandrus dispar*	Ås, Norway	PV350327	PV350327	PV330689	PV330697	PV330706
				NIBIO2016-1712/2/1	*Q. robur*/*Trypodendron domesticum*	Ås, Norway	PV350328	PV350328	PV330690	PV330698	PV330707
				KFL77916RJSR^D^	*Prunus domestica*/*Scolytus rugulosus*	Długołęka-Poland	MH283143	PV350329	MH283360	MH283499	MH283524
				KFL39616RJSI^D^	*Quercus robur*/*Scolytus intricatus*	Wierzchosławice, Poland	MH283142	PV350330	MH283359	MH283498	MH283523
		CBS 149830^C^	CMW 60554	KFL12WRJXM^D^	*Quercus robur*/*Xyleborus monographus*	Prószków, Poland	MH283141	PV350331	MH283357	MH283497	MH283522
		CBS 149831	CMW 60555	KFL121LMD^D^	*Larix decidua*/*Ips cembrae*	Rudziniec, Poland	KY568169	PV350332	KY568474	KY568674	MH283546
		CBS 149832	CMW 60556	KFL104716RJXS^D^	*Fagus sylvatica*/*Xyleborinus saxesenii*	Resko, Poland	MH283145	PV350333	MH283362	PV330699	MH283525
*Sporothrix fusiformis*	*Sporothrix* sp. 9	CBS112912^T^	CMW 9968		*Populus nigra*	Azerbaijan	AY280481	OM514870	AY280461	OM631912	JQ511967
		CBS 149833	CMW 60557	KFL104416RJAD^D^	*Quercus robur*/*Anisandrus dispar*	Resko, Poland	MH283159	PV350334	MH283381	MH283511	MH283540
		CBS 149834	CMW 60558	KFL103616RJAD^D^	*Quercus robur*/*Anisandrus dispar*	Resko, Poland	MH283158	PV350335	MH283379	MH283510	MH283538
		CBS 149835	CMW 60559	KFL56916RJAD^D^	*Quercus robur*/*Anisandrus dispar*	Resko, Poland	MH283154	PV350336	MH283375	MH283507	MH283535
				KFL57016RJAD^D^	*Quercus robur*/*Anisandrus dispar*	Resko, Poland	MH283155	PV350337	MH283376	MH283508	MH283536
*Spotothrix lunata*		CBS 112927^T^	CMW 10563		*Carpinus betulus*	Austria	AY280485	MH874477	AY280466	OM631918	JQ511970

^A^CBS = Westerdijk Fungal Biodiversity Institute, Utrecht, The Netherlands; CMW = Culture Collection of the Forestry and Agricultural Biotechnology Institute (FABI), University of Pretoria, Pretoria, South Africa; KFL = Culture collection of the Department of Forest Pathology, Mycology and Tree Physiology; University of Agriculture in Kraków, Poland; NIBIO = the culture collection of Norwegian Institute of Bioeconomy, Norway. ^B^ITS = internal transcribed spacer region of the nuclear ribosomal DNA gene; LSU = internal transcribed spacer region 2 and the 28S large subunit of the rDNA gene; *TUB*2 = β-tubulin; *TEF*1 = Translation elongation factor 1-alpha; *CAL*= calmodulin. Sequences with accession numbers PV350321-PV350337 were deposited in GenBank after concatenation of ITS and LSU sequences. ^C^ additional specimen examined. ^D^ Isolates collected during previous surveys in Poland and identified as *Sporothrix* sp. 4 and *Sporothrix* sp. 9 (Jankowiak et al. 2017, 2019b). ^T^ denotes ex-type cultures.

### ﻿PCR, sequencing and phylogenetic analyses

DNA extraction, PCR and sequencing were performed as described by Jankowiak et al. (2019a). For DNA sequencing and phylogenetic analyses, five loci were amplified as follows: the internal transcribed spacer regions ITS1-5.8S-ITS2 (ITS), the partial 28S ribosomal large subunit (LSU), the partial beta-tubulin (*TUB*2), the partial calmodulin (*CAL*) and the partial translation elongation factor 1-alpha (*TEF*1). The primers used for polymerase chain reaction (PCR) and sequencing of the various gene regions were as follows: ITS1-F (Gardes and Bruns 1993) and ITS4 (White et al. 1990) for ITS, LR5 and LR0R (Vilgalys and Hester 1990) for LSU, Bt2a and Bt2b (Glass and Donaldson 1995) for *TUB*2, EF2F (Marincowitz et al. 2015) and EF2R (Jacobs et al. 2004) or F-728F (Carbone and Kohn 1999) and EF2 (O’Donnell et al. 1998) for *TEF*1 and CL1 and CL2a (O’Donnell et al. 2000) or CL3F and CL3R (De Beer et al. 2016) for *CAL*.

**Figure 1.**
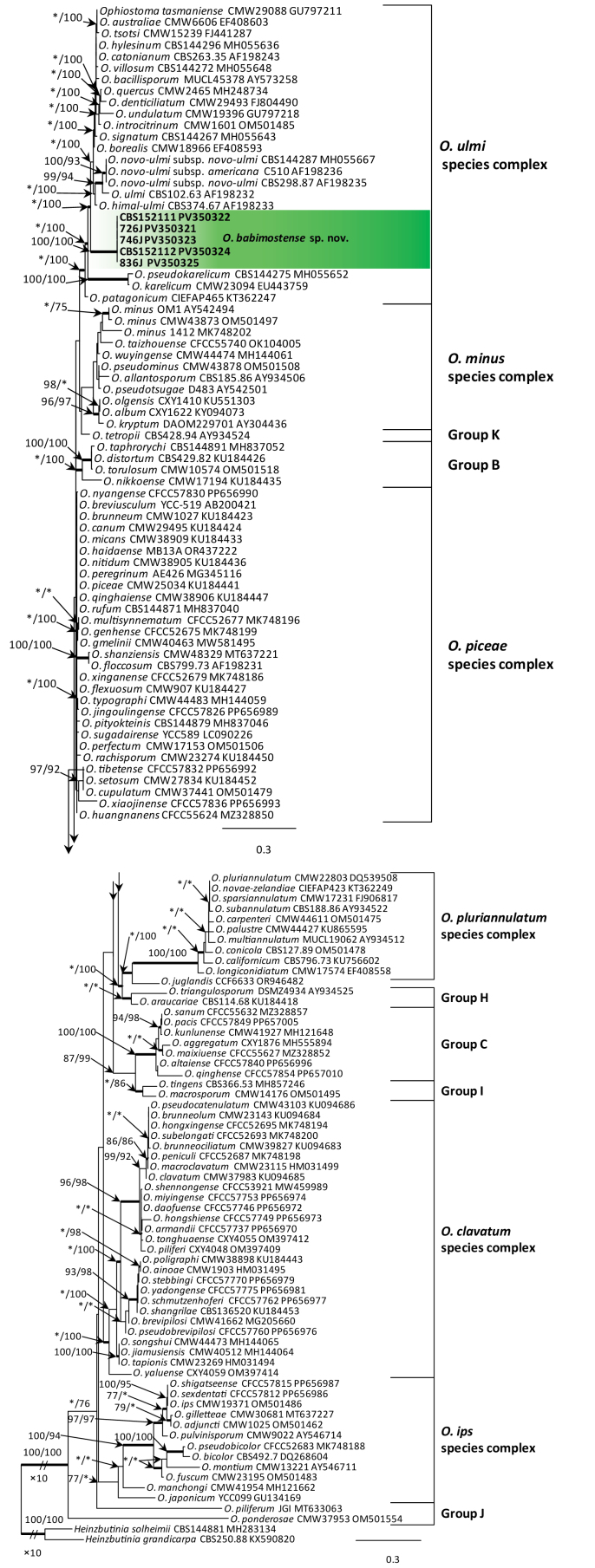
Phylogram from Maximum Likelihood (ML) analysis of ITS data for *Ophiostoma* spp. Polish isolates used in this study are in bold. Polish isolates used in this study are in bold. Bootstrap values (if ≥ 75%) for ML and Maximum Parsimony (MP) analyses are presented at the nodes as follows: ML/MP. Bold branches indicate posterior probabilities values ≥ 0.95 obtained from Bayesian Inference (BI) analysis. * Bootstrap values < 75%. The tree is drawn to scale (see bar) with branch lengths measured in the number of substitutions per site. The species complexes were designated, based on the classification of De Beer et al. (2022) and Wang et al. (2024). *Heinzbutinia solheimii* and *H. grandicarpa* represent the outgroup.

**Figure 2.**
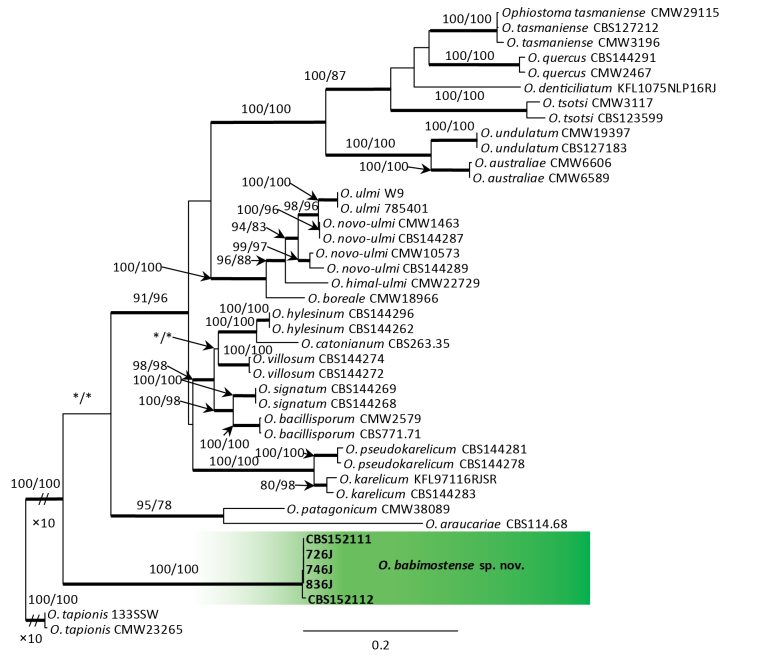
Phylogram from Maximum Likelihood (ML) analysis of the combined datasets of *TUB*2+*TEF*1 for the *Ophiostoma ulmi* species complex. Polish isolates used in this study are in bold. Bootstrap values (if ≥ 75%) for ML and Maximum Parsimony (MP) analyses are presented at the nodes as follows: ML/MP. Bold branches indicate posterior probabilities values ≥ 0.95 obtained from Bayesian Inference (BI) analysis. * Bootstrap values < 75%. The tree is drawn to scale (see bar) with branch lengths measured in the number of substitutions per site. *Ophiostoma tapionis* represents the outgroup.

**Figure 3.**
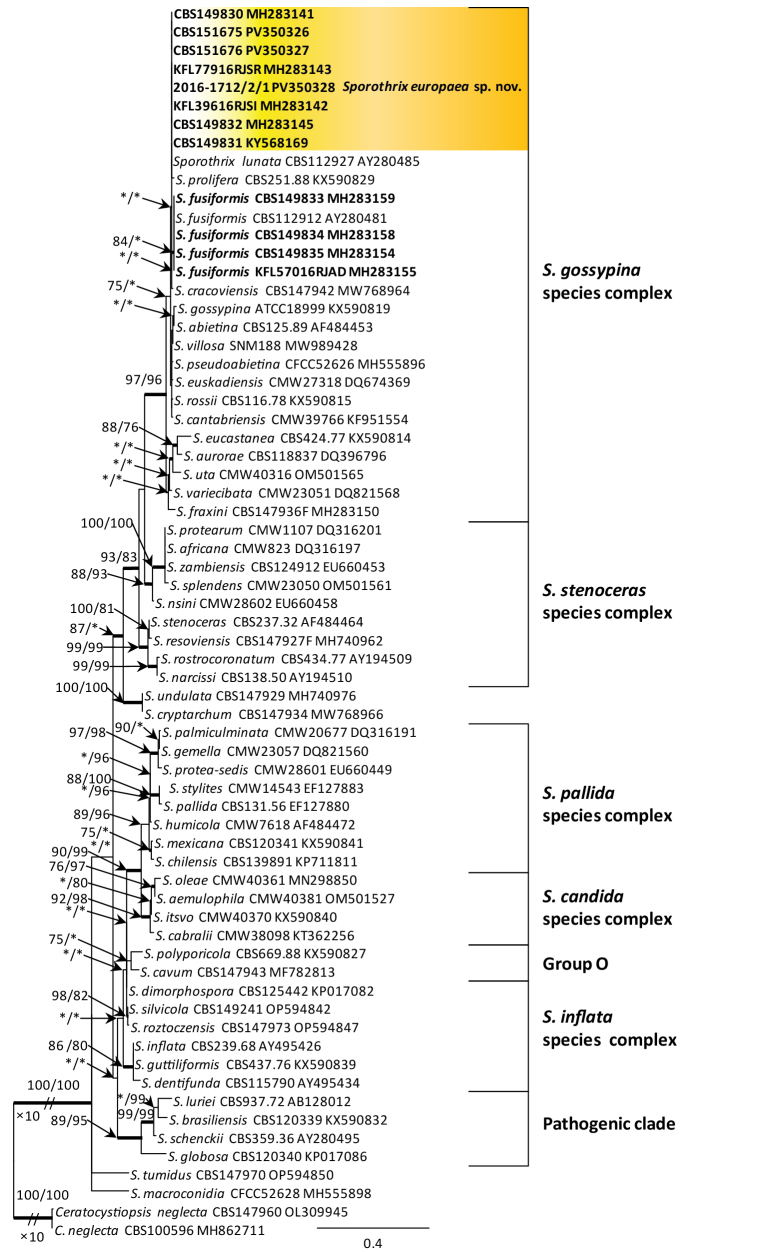
Phylogram from Maximum Likelihood (ML) analysis of ITS data for *Sporothrix* spp. Norwegian and Polish isolates used in this study are in bold. Bootstrap values (if ≥ 75%) for ML and Maximum Parsimony (MP) analyses are presented at the nodes as follows: ML/MP. Bold branches indicate posterior probabilities values ≥ 0.95 obtained from Bayesian Inference (BI) analysis. * Bootstrap values < 75%. The tree is drawn to scale (see bar) with branch lengths measured in the number of substitutions per site. The species complexes were designated, based on the classification of De Beer et al. (2016). *Ceratocystiopsis neglecta* represents the outgroup.

**Figure 4.**
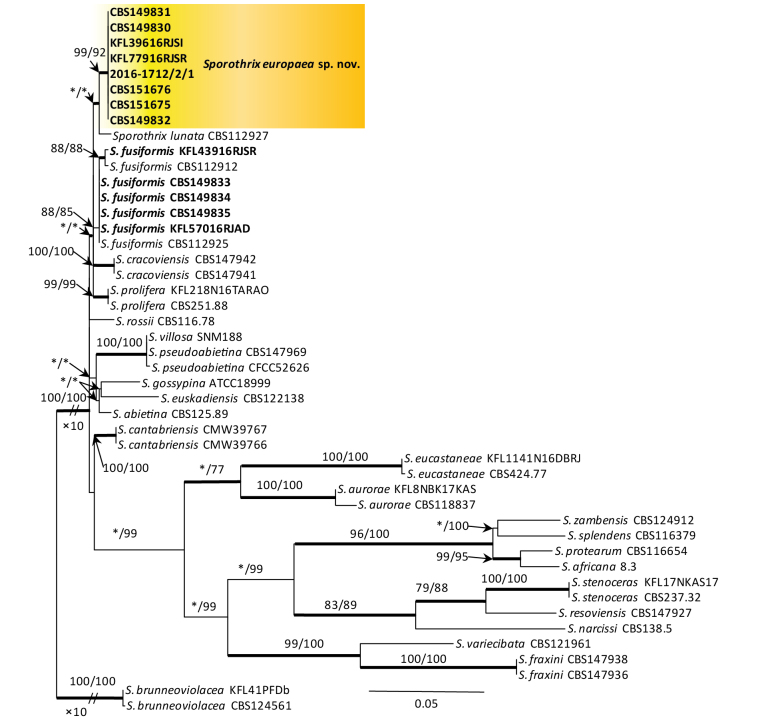
Phylogram from Maximum Likelihood (ML) analysis of the combined datasets of *TUB*2+*CAL* for the *Sporothrix stenoceras* and *S. gossypina* complexes. Norwegian and Polish isolates used in this study are in bold. Bootstrap values (if ≥ 75%) for ML and Maximum Parsimony (MP) analysis are presented at the nodes as follows: ML/MP. Bold branches indicate posterior probabilities values ≥ 0.95 obtained from Bayesian Inference (BI) analysis. * Bootstrap values < 75%. The tree is drawn to scale (see bar) with branch lengths measured in the number of substitutions per site. *Sporothrix brunneoviolacea* represents the outgroup.

BLAST searches using the BLASTn algorithm were performed to retrieve similar sequences from GenBank (http://www.ncbi.nlm.nih.gov) and accession numbers for these sequences are presented in the corresponding phylogenetic trees (Figs 1–4). Datasets were curated using Molecular Evolutionary Genetic Analysis (MEGA) 6.06 (Tamura et al. 2013). The ITS datasets included all available sequences for reference species of *Ophiostoma* and *Sporothrix* that could be retrieved from GenBank to resolve the placement of the isolates within the mentioned genera.

Sequence alignments were performed using the online version of MAFFT 7 (Katoh and Standley 2013) using the E-INS-i strategy with a 200PAM/κ = 2 scoring matrix, a gap opening penalty of 1.53 and an offset value of 0.00. The alignments were checked manually with BioEdit 2.7.5 (Hall 1999). The resulting alignments and trees were deposited in TreeBASE (http://purl.org/phylo/treebase/phylows/study/TB2:S32067).

All phylogenetic analyses were performed independently for each gene regions (ITS, *TUB*2, *TEF*1 for *Ophiostoma* and ITS, *CAL*, *TUB*2 for *Sporothrix*). Sequences of the LSU (for *Ophiostoma*, *Sporothrix*), *CAL* (for *Ophiostomа*) and *TEF*1 (for *Sporothrix*) genes were generated for future reference. Gene regions showing no conflicts in the grouping of isolates at the terminal clades (i.e. *TUB*2 and *TEF*1 for *Ophiostoma* and *TUB*2 and *CAL* for *Sporothrix*), were combined and analysed as concatenated datasets.

Phylogenetic trees were inferred for each of the datasets using three different methods: Maximum Likelihood (ML), Maximum Parsimony (MP) and Bayesian Inference (BI). For ML and BI analyses, the best-fit substitution models for each dataset were determined using the corrected Akaike Information Criterion (AICc) in jModelTest 2.1.10 (Guindon and Gascuel 2003; Darriba et al. 2012). ML analyses were carried out using PhyML 3.0 (Guindon et al. 2010), utilising the Montpelier online server (http://www.atgc-montpellier.fr/phyml/). The ML analysis included bootstrap analysis (1000 bootstrap pseudoreplicates) in order to assess node support values.

MP analyses were performed using PAUP* 4.0b10 (Swofford 2003). Gaps were treated as the fifth state. Bootstrap analysis (1000 bootstrap replicates) was conducted to determine the levels of confidence for the nodes within the inferred tree topologies. Tree bisection and reconnection (TBR) was selected as the branch swapping option. The tree length (TL), consistency index (CI), retention index (RI), homoplasy index (HI) and rescaled consistency index (RC) were recorded for each analysed dataset after the trees were generated.

BI analyses using Markov Chain Monte Carlo (MCMC) methods were carried out using MrBayes 3.1.2 (Ronquist and Huelsenbeck 2003). Four MCMC chains were run for 10 million generations, applying the best-fit model for each dataset. Trees were sampled every 100 generations, resulting in 100,000 trees. Tracer 1.4.1 (Rambaut and Drummond 2007) was utilised to determine the burn-in value for each data-set. The remaining trees were utilised to generate a 50% majority rule consensus tree, which allowed for calculating posterior probability values for the nodes.

### ﻿Morphology and growth rate

Morphological characters were examined for selected isolates as well as for the herbarium specimens selected as types. Cultures were grown on 2% malt extract agar (MEA) made up of 20 g malt extract, 20 g bacteriological lab-agar (Biomaxima S.A., Lublin, Poland) in 1 litre deionised water. Fungal cultures were derived from single conidia spores. The cultures were incubated at 25 °C and monitored regularly for the appearance of sporulating structures.

Attempts were made to induce the formation of sporulating structures, by placing autoclaved twigs of host trees at the centres of agar plates containing 2% MEA. To promote the production of ascomata, all isolates of each species were crossed in all possible combinations, following the technique described by Grobbelaar et al. (2009). These cultures were incubated 25 °C in darkness and monitored regularly for the appearance of sporulating structures.

Morphological features were examined by mounting fungal tissue in 80% lactic acid on glass slides and fruiting structures were observed using a Nikon Eclipse 50i microscope (Nikon, Tokyo, Japan) with an Invenio 5S digital camera (DeltaPix, Maalov, Denmark) to capture photographic images. Colour designations were based on the colour charts of Kornerup and Wanscher (1978).

For each taxonomically relevant structure, 50 measurements were made, when possible, using Coolview 1.6.0 (Precoptic, Warsaw, Poland). Averages, ranges and standard deviations were calculated for the measurements and these are presented in the format “(min–)(mean − SD)–(mean + SD)(–max)”.

Growth characters for the novel species were determined by analysing the growth for four isolates, including two for each species (Table 1). Agar disks (5 mm diam.) were cut from the actively growing margins of fungal colonies and these discs were placed at the centres of plates containing 2% MEA. Four replicate plates for each of the two putative new species were incubated at temperatures between 5 and 35 °C, at 5 °C intervals. The mean colony diameter and the radial growth (two measurements perpendicular to each other per plate) were determined 7 and 14 d after inoculation and growth rates were calculated as mm/d.

## ﻿Results

### ﻿DNA sequence data and phylogenetic analysis

DNA sequence data were generated for 13 isolates considered in this study (Table 1). BLAST analyses of the ribosomal DNA sequences and phylogenetic analyses of the ITS sequences placed five isolates within *Ophiostoma* and eight isolates within *Sporothrix* (Figs 1, 3).

The best evolutionary substitution models identified for the *Ophiostoma* datasets were as follows: GTR+I+G for ITS (−lnL = 7360.57), HKY+I+G for *TUB*2 (−lnL = 2541.70), GTR+I+G for *TEF*1 (−lnL = 5638.21) and GTR+I+G for the combined *TUB*2+*TEF*1 dataset (−lnL = 8370.00). For the *Sporothrix* datasets, the best substitution models were as follows: GTR+I+G for ITS (−lnL = 3664.91), GTR+G for *TUB*2 (−lnL = 1474.17), GTR+I+G for *CAL* (−lnL = 3780.98) and GTR+G for the combined *TUB*2+CAL dataset (−lnL = 5380.69).

#### ﻿*Ophiostoma*

Alignments for the ITS dataset contained 796 characters, while *TUB*2 and *TEF*1 had 396 and 731 characters, respectively. The combined *TUB*2+*TEF*1 dataset included 1127 characters. Of these, 297 characters in the ITS dataset, 150 in *TUB*2, 394 in *TEF*1 and 554 in the *TUB*2+*TEF*1 dataset were parsimony-informative. The aligned the *TUB*2 data used for phylogenetic analyses included exons 4, 5 and 6, interspersed by introns 3 and 4. The aligned the *TEF*1 data used for phylogenetic analyses included exons 4 and 5, preceded by intron 3.

The analyses of ITS sequences (Fig. 1) showed that five isolates grouped closest to the members of the *O. ulmi* species complex and were distinct from all other species in this species complex. These isolates formed distinct clade with high statistical supports. In the *TUB*2 and *TEF*1 trees (Suppl. material 1: figs S1, S2), these isolates formed well-supported lineages that clearly separated this newly-proposed species from all the other known species in the *O. ulmi* species complex. The combined analyses of the *TUB*2 and *TEF*1 datasets (Fig. 2) also showed that these isolates formed a distinct and well-supported clade within the *O. ulmi* complex.

#### ﻿*Sporothrix*

The ITS dataset consisted of 631 characters. For the individual gene regions, *TUB*2 had 364 characters and *CAL* had 825 characters. When *TUB*2 and *CAL* were combined (*TUB*2+*CAL*), the dataset contained 1189 characters. Regarding parsimony-informative characters, the breakdown was as follows: 210 in the ITS dataset, 87 in *TUB*2, 266 in *CAL* and 353 in the combined *TUB*2+*CAL* dataset. Alignments for the ITS dataset contained 631 characters and for *TUB*2+*CAL* 1189 characters, of which 210 and 353 were parsimony informative, respectively. The aligned the *TUB*2 data used for phylogenetic analyses included exons 4, 5 and 6, interspersed by introns 4 and 5. The aligned the *CAL* data used for phylogenetic analyses included exons 3, 4, 5 and 6, interspersed by introns 3, 4 and 5.

The analyses of ITS sequences showed that 13 isolates, labelled as *Sporothrix* sp. 4 and *Sporothrix* sp. 9 by Jankowiak et al. (2019b), resided in the *S. gossypina* species complex and were not distinct from some other species in this species complex (Fig. 3). Based on the *TUB*2 phylogeny, eight isolates of *Sporothrix* sp. 4 grouped closely with *S. fusiformis*. Based on the *CAL* phylogeny, isolates of *Sporothrix* sp. 4 formed a distinct and well-supported clade which was close to, but distinct from *S. lunata* (Suppl. material 1: figs S3, S4). The combined analyses of the *TUB*2 and *CAL* datasets showed that eight isolates of *Sporothrix* sp. 4 formed a well-resolved clade and this formed a sister clade with *S. lunata* (Fig. 4). Analyses of *TUB*2 and *CAL* sequences and the combined analyses of these both genes clearly showed that four isolates of *Sporothrix* sp. 9 belonging to known species, *S. fusiformis* (Fig. 4).

### ﻿Taxonomy

#### ﻿ Ophiostoma babimostense

Taxon classificationFungiOphiostomatalesOphiostomataceae

R. Jankowiak sp. nov.

82804642-68E5-54AA-98C6-A5862372A6F8

858470

Fig. 5

##### Etymology.

The specific epithet “babimostense” refers to the name of locality in Poland, Babimost, where the fungus was isolated for the first time.

**Figure 5.**
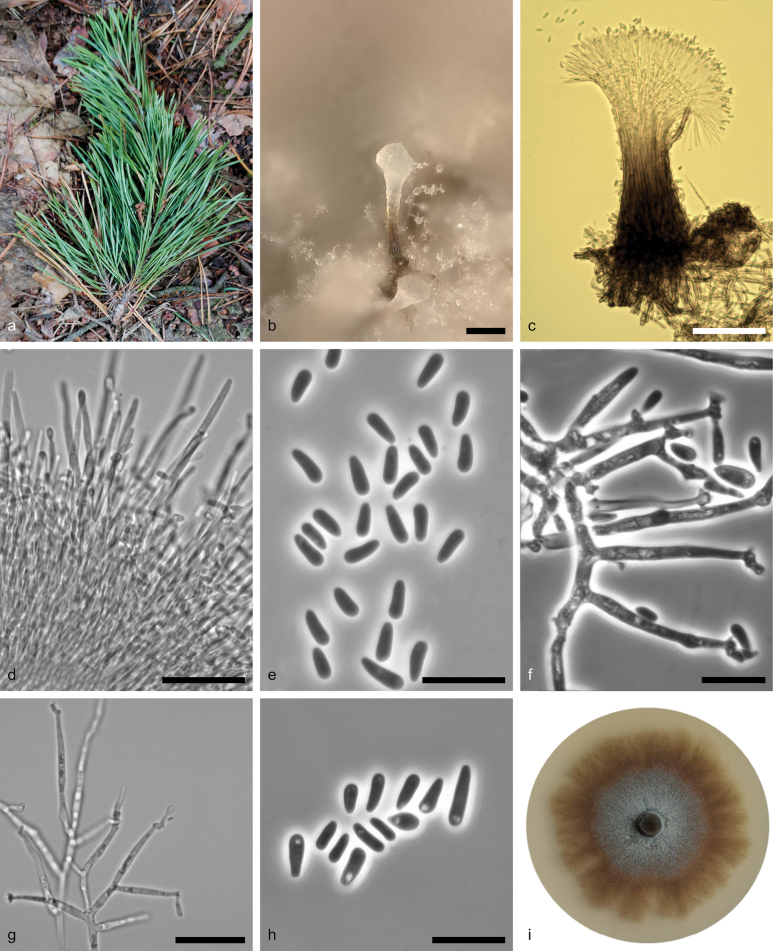
*Ophiostoma babimostense* sp. nov. (CBS 152112). a. Fallen shoot of Scots pine pruned by *Tomicus* spp.; b, c. Synnemata in culture; d. Conidiogenous apparatus showing conidiogenous cells; e. Conidia; f, g. Mononematous, macronematous conidiophores with apical denticles; h. Conidia; i. Fourteen-day-old culture on MEA. Scale bars: 50 μm (b–c); 25 μm (d); 10 μm (e–f); 25 μm (g); 10 μm (h).

##### Type.

Poland • Lubuskie Province, Babimost, fallen shoots of *Pinus sylvestris* pruned by pine shoot beetles, *Tomicus* sp., October 2023, *coll.* R. Jankowiak (holotype KRAM F-60035, ex-type culture CBS 152112).

##### Description.

***Sexual morph***: not observed. ***Asexual morphs*** synnematous, pesotum-like and mononematous to micronematous (sporothrix-like type). ***Synnemata*** abundant on MEA and sterilised pine twigs, determinate, erect, single or in groups, arising from the agar, pine twigs or aerial mycelium and attached to substratum by brown, rhizoid-like hyphae, dark brown to black and becoming subhyaline to hyaline towards the conidiogenous apparatus, (127–)145.5–239(–315) μm long including the capitulum. ***Stipe*** dark brown to black at the bases, light brown or yellowish-brown at the centre and hyaline at the apex, (60–)86–153.5(–210) μm long, broadest towards the base, (16.5–)24–48.5(–81.5) µm down to (11.5–)18.5–44(–91) µm wide at the apex, cylindrical, smooth. ***Conidiophores*** branching divaricate or dichotomous with 2 (mostly) or 3 conidiogenous cells per branch point. ***Conidiogenous cells*** annellated, discrete, terminal, cylindrical, tapering towards apex, hyaline, smooth, (16.5–)27–42.8(–50) × (0.8–)0.9–1.5(–2) μm with. ***Conidia*** aseptate, hyaline, curved, obovate, (3–)3.5–4.5(–6) × (1–)1.5–2(–2.5) µm, accumulating in a terminal mucilagenous mass, hyaline, transparent and glassy when young, becoming white with age. Sporothrix-like type: ***conidiophores*** macronematous to micronematous, hyaline, produced as aerial mycelia, simple or irregularly or dichotomous branched, producing conidia from denticles in a sporothrix-like fashion. ***Conidiogenous cells*** hyaline, smooth, straight or curved, integrated or discrete, terminal or intercalary, cylindrical, tapering towards apex, (18.5–)20–43.5(–73.5) × (1–)1.5–2.5(–3) µm, apex becoming nodose from numerous denticles, often proliferating at the apex and giving rise to another nodose or a conidiogenous cell with nodose at the apex. ***Conidia*** solitary, abundant in cultures, more varied to the size and shape of conidia produced in synnemata, hyaline, aseptate, smooth, oblong, obovate, (3–)4–5.5(–8) × (1–)1.5–2(–3) µm.

##### Culture characteristics.

*Colonies* with optimal growth at 25 °C on 2% MEA, reaching 66 mm (± 0.59 mm) diam. in 14 d, with a radial growth rate of 0.24 mm/d, followed by 20 °C (55 mm, ± 0.21 mm) diam. Colony olive-brown (1E5), with pale grey (1B1) aerial mycelia at the centre; with age, colonies become olive-grey (1E2); flat, with undulate margin, revers olive (1F4). Hyphae pale yellow (1A3) to olive-yellow (3E6) in colour (Kornerup and Wanscher 1978), smooth, with or without granules, submerged in the medium and aerial mycelium abundant, often constricted at the septa, (1–) 1.5–2.5 (–3) µm wide.

##### Associated insect.

*Tomicus* spp.

##### Host tree.

*Pinus sylvestris*

##### Distribution.

Poland

##### Additional material examined.

Poland • Lubuskie Province, Babimost, from fallen shoots of *Pinus sylvestris* pruned by pine shoot beetles, *Tomicus* sp., October 2023, *coll. R. Jankowiak* (culture CBS 152111).

##### Notes.

The newly-described *O. babimostense* resides in the *O. ulmi* species complex. *Ophiostoma babimostense* is morphologically similar to many species of the *O. ulmi* complex. However, *O. babimostense* does not form a monophyletic clade with other species in the *O. ulmi* complex (Figs 1, 3, Suppl. material 1: figs S1, S2). This species fails to group consistently with any one member of the *O. ulmi* complex within the ITS- (Fig. 1), *TUB*2- (Suppl. material 1: fig. S1) and *TEF*1- (Suppl. material 1: fig. S3) based trees. In addition, *O. babimostense* formed a distinct lineage basal to all other species in the *O. ulmi* complex in the combined analyses of the *TUB*2 and *TEF*1 datasets (Fig. 3).

#### ﻿ Sporothrix europaea

Taxon classificationFungiOphiostomatalesOphiostomataceae

R. Jankowiak & H. Solheim sp. nov.

DA31E7C6-F903-5CE7-B64E-43D831626355

Mycobank No: 858471

Fig. 6

##### Etymology.

The specific epithet “*europaea*” (Latin) refers to the European continent, where this fungus was isolated in Norway and Poland.

**Figure 6.**
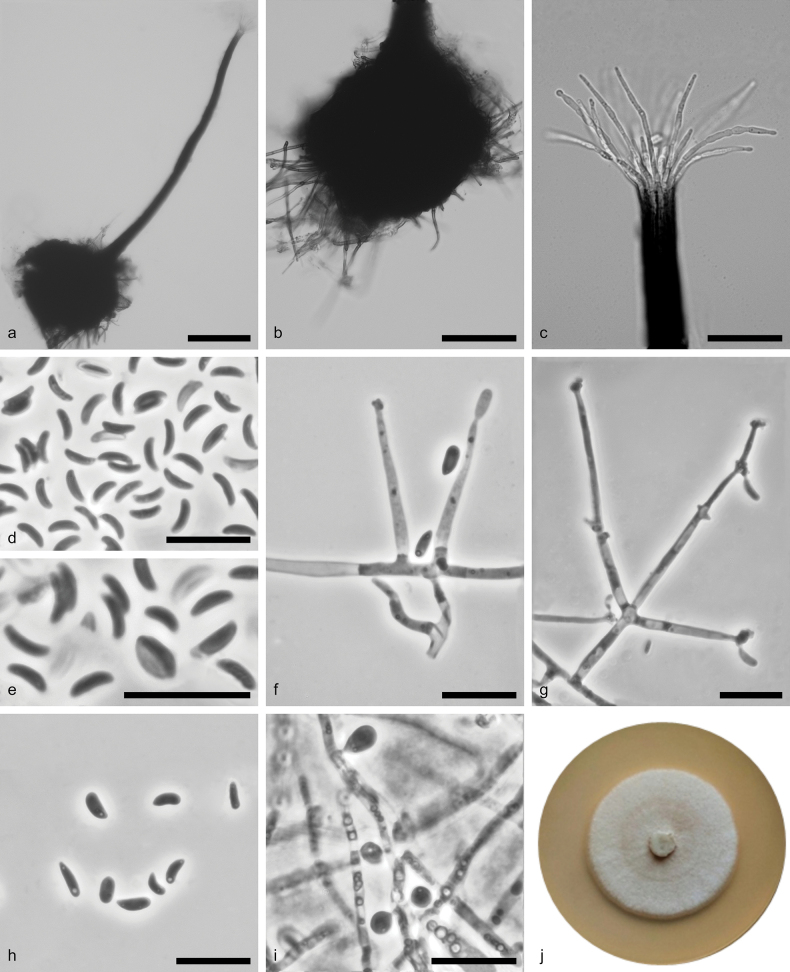
*Sporothrix europaea* sp. nov. (CBS 151676). a. Ascoma; b. Ascomatal base; c. Ostiolar hyphae; d, e. Ascospores; f, g. Conidiogenous cell with an inflated cluster of denticles at the apex; h. Conidia; i. Globose conidia arising directly from hyphae; j. Fourteen-day-old culture on MEA. Scale bars: 100 (a); 50 μm (b); 25 μm (c); 10 μm (d–i).

##### Type.

Norway • Ås, from *Anisandrus dispar* infesting *Quercus robur*, June 2016, *coll.* T. Aas, (holotype KRAM F-60036, culture ex-type CBS 151676).

##### Description.

Sexual and asexual morphs produced on the sterilised beech twigs and on the surface of malt agar in Petri dishes. ***Sexual morphs***: ***ascomata*** perithecial, abundant, superficially or partly embedded in the agar, single or in groups. ***Perithecial bases*** globose, (86–) 105–160 (–193) μm diam., black, with brown hyphal hairs, 30–84 μm long and 1.3–2.4 μm wide at the base. ***Perithecial necks*** black, straight or curved, (257–)336–456(–530) μm long, (8.5–)9.5–13(–14.5) μm diam. at the apex and (22–)26–39(–57.5) μm at the base. ***Ostiolar hyphae*** present, pale brown, septate, straight or curved, simple, tips blunting or strongly thickened, 10–19 in number, (14–)29–42.5(–48.5) μm long, 0.5–1.5 μm at the apex and 1–2.5 μm at the base. ***Asci*** evanescent. ***Ascospores*** one-celled, hyaline, allantoid in side view (3–)3.5–4(–5) x (0.5–)1–1.5(–2) μm, elliptical in front view (2.5–)3–4(–5) × (1–)1–1.5(–1.5) μm, sometimes with residual sheath up to 2 μm thick, accumulated in white-colour mass at the tip of the neck. ***Asexual morph***Sporothrix-like: ***conidiophores*** hyaline, micronematous, simple or branched and bearing several conidiogenous cells, borne on upright undifferentiated hyphae. ***Conidiogenous cells*** cylindrical, terminal or intercalary, straight or curved, tapering towards the apex, (3.5–)15–36(–50.5) μm long, (0.5–)1–2(–2) μm wide at the base, the apical part swollen, (1–)2–3(–3) μm long, (1.5–)2–4(–5.5) μm wide, with multiple conidiogenous loci as denticles, born by sympodial proliferation. ***Conidia*** of two types: 1) abundant in cultures, hyaline, unicellular, smooth, variable in shape and size, guttuliform to fusiform, curved, often asymmetric, pointed at the base, (3–)3.5–5(–7) × (0.5–)1–1.5(–2.5) μm, formed directly on denticles; 2) sparse in cultures, directly on the side of submerged hyphae in malt agar, subhyaline to lightly pigmented, unicellular, smooth, subglobose to broadly obovate, (2.5–)3–4(–5.5) × (2–)2–3(–4) μm diam., formed singly.

##### Culture characteristics.

Colonies with optimal growth at 25 °C on 2% MEA reaching an average of 47 mm (± 0.07 mm) after 14 days, with radial growth rate 1.68 (± 0.24) mm/d, growth somewhat slower at 30 °C (40 mm diameter); white (3A1), flat, floccose, growing in a circular pattern with entire margins, reverse yellowish-white (3A2). Hyphae greenish-grey (1B7) in colour, smooth, with granules, submerged in the medium and aerial mycelium abundant, (0.5–)1–1.5(–3) µm wide.

##### Associated insects.

*Anisandrus dispar*, *Ips cembrae*, *Scolytus intricatus*, *Scolytus rugulosus*, *Xyleborinus saxesenii*, *Xyleborus monographus*.

##### Host trees.

*Fagus sylvatica*, *Larix decidua*, *Prunus domestica*, *Quercus robur*

##### Distribution.

Norway, Poland

##### Additional specimen examined.

Poland • Prószków, from *Xyleborus monographus* infesting *Quercus robur*, May 2013, coll. P. Wieczorek, (culture CBS 149830=CMW 60554); Norway • Ås, *Anisandrus dispar* on *Quercus robur*, June 2016, *coll.* T. Aas, (culture CBS 151675).

##### Notes.

This species is phylogenetically distinct from the other *Sporothrix* species, based on phylogenetic analysis of combined *TUB*2 and *CAL* sequence data (Fig. 4). *Sporothrix europaea* is phylogenetically closely related to *S. fusiformis* and *S. lunata* described by Aghayeva et al. (2004). However, *S. europaea* has smaller ascomatal bases and necks compared to *S. fusiformis* (86–193 μm and 257–530 μm vs. 121–273 μm and 301–1168 μm). In turn, *S. europaea* has larger ascomatal bases (86–193 μm vs. 60–178 μm) and longer necks (257–530 μm vs. 162–700 μm) compared to *S. lunata* (Aghayeva et al. 2004). In addition, *S. europaea* has two conidial types, guttuliform to fusiform and subglobose to obovate, whereas those in cultures of *S. fusiformis* are only guttuliform to fusiform.

*Spotothrix lunata* has lunate conidia flattened at one side or curved with a blunt base (Aghayeva et al. 2004). The cultures of *S. europaea* grow on 2% MEA between 5 °C and 35 °C, whereas *S. fusiformis* and *S. lunata* did not growth below 10 °C and above 30 °C (Aghayeva et al. 2004).

*Sporothrix europaea* was represented by five isolates collected from Poland. It corresponds to *Sporothrix* sp. 4 in the study of Jankowiak et al. (2019b). *Sporothrix europaea* was isolated from hardwoods in association with different bark beetles.

## ﻿Discussion

In this study, a set of ophiostomatalean isolates from Norway and Poland were shown to represent two new taxa in the Ophiostomatales (Ascomycota), *Ophiostoma babimostense* and *Sporothrix europaea*.

Morphological comparisons and multigene phylogenies placed *O. babimostense* as an additional species within the *O. ulmi* species complex. The new species described herein exhibit morphological resemblance to the other members of the *O. ulmi* complex producing pesotum- and sporothrix-like morphs. Most of the members of the complex produce ascomata with long necks and allantoid ascospores that lack sheaths (De Beer and Wingfield 2013; De Beer et al. 2022). *Ophiostoma babimostense* is not easily distinguished morphologically from other members of the *O. ulmi* species complex, especially regarding morphology of the asexual morph. However, it is different from most members of the *O. ulmi* complex by the lack of a sexual morph in culture. Currently no sexual morphs are known for *O. hylesinum* and *O. australiae*. The first species was described from *Fraxinus excelsior* L. infested by bark beetles (Aas et al. 2018), while *O. australiae* was isolated from wounds on *Acacia mearnsii* De Wild. trees in Australia (Kamgan Nkuekam et al. 2008).

Twenty-one known species were included previously in the *O. ulmi* complex (De Beer et al. 2022). The *O. ulmi* complex includes species what was previously referred to as the ‘hardwood clade’ in the *O. piceae* complex (Harrington et al. 2001; Linnakoski et al. 2010). It was later considered as the *O. quercus* complex (Kamgan Nkuekam et al. 2011). Finally, the *O. ulmi* species complex has been defined by De Beer and Wingfield (2013). The discovery of *O. babimostense* known hitherto only from conifers was unexpected because members of this complex had been, so far, only found in association with a variety of bark beetles on hardwoods. Nonetheless, recently, two other members of the *O. ulmi* complex, namely *O. borealis* and *O. pseudokarelicum*, have also been recorded from conifer hosts in the western Carpathian (Jankowiak et al. 2017). Furthermore, *O. quercus*, a widespread species that primarily infects sapstain in hardwood hosts (Taerum et al. 2018), has been isolated also from conifers (Reay et al. 2005; Thwaites et al. 2005; Zhou et al. 2006; Jankowiak 2012; Jankowiak and Bilański 2013a, 2013b; Jankowiak et al. 2017). It seems that some species of the *O. ulmi* complex can be found in a much broader range of hosts than what is commonly considered and host specificity is not as strict for some species.

*Ophiostoma babimostense* has been isolated from Scots pine shoots infested by an unknown species of *Tomicus*, probably *T. piniperda* (L.) or *T. minor* (Hart.). This species was not observed in previous studies dealing with associations of Scots pine-infesting *Tomicus* spp. and fungi in Poland (Jankowiak 2006, 2008; Jankowiak and Bilański 2007), but was reported as an unknown species later on (Jankowiak and Kolařík 2011; labelled in this paper as *Ophiostoma* sp. 1). The ecological niche of *O. babimostense* remains incompletely unknown. It can be assumed that it is associated with *Tomicus* species in Poland although this should be confirmed.

*Sporothrix europaea* forms part of the *S. gossypina* complex, bringing the total number of species in the complex to 19. This species complex was introduced by De Beer et al. (2016). Members of the *S. gossypina* complex are most often associated with bark and ambrosia beetles, although a few species have been isolated from stained oak wood or tree wounds (Aghayeva et al. 2004; De Beer et al. 2022).

*Sporothrix europaea* was isolated from different beetle species and host trees (Jankowiak et al. 2017, 2019b; labelled in this paper as *Sporothrix* sp. 4), confirming that it is not host specific. In a Polish study, *S. europaea* was found in association with *Scolytus intricatus* and *Xyleborus monographus* on *Quercus robur*, *Scolytus rugulosus* on *Prunus domestica* and *Anisandrus dispar* and *Xyleborinus saxesenii* on *Fagus sylvatica* (Jankowiak et al. 2019b; Suppl. material 1). In addition, a single isolate of *S. europaea* was isolated from *Ips cembrae* on *Larix decidua* (Jankowiak et al. 2017). The Norwegian isolates of *S. europaea* were collected from *Q. robur* infested by *A. dispar* and *Trypodendron domesticum* (H. Solheim, unpublished data).

In addition, our data show that *S. fusiformis* is also commonly associated with diverse species of bark beetles and host trees, including *A. dispar*, *Dryocoetes villosus*, *S. intricatus*, *S. ratzeburgi*, *X. monographus*, *X. saxesenii* and *Betula pendula*, *Fagus sylvatica*, *Larix decidua*, *Prunus domestica* and *Quercus robur* (Jankowiak et al. 2019b; species labelled in this paper as *Sporothrix* sp. 9). This fungus has previously been described by Aghayeva et al. (2004), where it was found in Azerbaijan and Austria occurring on stained wood of *Castanea sativa*, *L. decidua*, *Populus nigra*, *Quercus petraea* (Aghayeva et al. 2004). In addition, *S. fusiformis* was previously found in association with *Ips cembrae* in Austria (Aghayeva et al. 2004).

Species of the *S. gossypina* complex exhibit a relative morphological homogeneity. Based on DNA sequence comparisons, *S. europaea* is closely related to *S. fusiformis* and *S. lunata*. In addition to phylogenetic differences, these species can be separated from each other, based on characteristics of their sexual and asexual morphs.

The present study provides novel information about the new species from the *O. ulmi* complex that includes non-pathogenic species like *Ophiostoma quercus* (Taerum et al. 2018) or the highly aggressive pathogenic fungus *O. novo-ulmi*, the causal agents of Dutch Elm Disease (DED) (Brasier 1991). The results of this study have also expanded our knowledge of *Sporothrix*. Broadly, the results suggest that *Sporothrix* species from the *S. gossypina* complex are common and ecologically diverse members of the Ophiostomatales in hardwood ecosystems in Europe.

## Supplementary Material

XML Treatment for Ophiostoma babimostense

XML Treatment for Sporothrix europaea

## ﻿Additional information

### Use of AI

No use of AI was reported.

### Author ORCIDs

Robert Jankowiak https://orcid.org/0000-0002-2804-5396

Piotr Bilański https://orcid.org/0000-0002-2750-6699

### Data availability

All of the data that support the findings of this study are available in the main text or Supplementary Information.

## Supplementary materials

Supplementary material 1 Supplementary figures Data type docx https://binary.pensoft.net/file/1436728This dataset is made available under the Open Database License (http://opendatacommons.org/licenses/odbl/1.0/). The Open Database License (ODbL) is a license agreement intended to allow users to freely share, modify, and use this Dataset while maintaining this same freedom for others, provided that the original source and author(s) are credited.Robert Jankowiak, Halvor Solheim, Piotr Bilański, Filip Kawa
