# Supplementary material for: ﻿Ophiostoma babimostense and Sporothrix europaea (Ascomycota, Ophiostomatales), two new ophiostomatalean species, associated with ambrosia and bark beetles in Norway and Poland
Source: MycoKeys. 2025 Oct 13;123:121–45. doi: 10.3897/mycokeys.123.155588 (PMC12538221; doi:10.3897/mycokeys.123.155588)
Supplement: Supplementary material 1 — Supplementary figures [file mycokeys-123-121-s001.docx]

**Figure S1.** Phylogram from Maximum Likelihood (ML) analysis of *TUB*2 data for the *Ophiostoma ulmi* species complex. Sequences obtained in this study are in bold. Bootstrap values (if ≥ 75%) for ML and Maximum Parsimony (MP) analyses are presented at the nodes as follows: ML/MP. Bold branches indicate posterior probabilities values ≥ 0.95 obtained from Bayesian Inference (BI) analysis. * Bootstrap values <75%. The tree is drawn to scale (see bar) with branch lengths measured in the number of substitutions per site. *Ophiostoma tapionis* represents the outgroup.

**Figure S2.** Phylogram from Maximum Likelihood (ML) analysis of *TEF*1 data for the *Ophiostoma ulmi* species complex. Sequences obtained in this study are in bold. Bootstrap values (if ≥ 75%) for ML and Maximum Parsimony (MP) analyses are presented at the nodes as follows: ML/MP. Bold branches indicate posterior probabilities values ≥ 0.95 obtained from Bayesian Inference (BI) analysis. * Bootstrap values <75%. The tree is drawn to scale (see bar) with branch lengths measured in the number of substitutions per site. *Ophiostoma tapionis* represents the outgroup.

**Figure S3.** Phylogram from Maximum Likelihood (ML) analysis of *TUB*2 data for the *Sporothrix stenoceras* & *S. gossypina* complexes. Norwegian and Polish isolates used in this study are in bold. Bootstrap values (if ≥ 75%) for ML and Maximum Parsimony (MP) analyses are presented at the nodes as follows: ML/MP. Bold branches indicate posterior probabilities values ≥ 0.95 obtained from Bayesian Inference (BI) analysis. * Bootstrap values <75%. The tree is drawn to scale (see bar) with branch lengths measured in the number of substitutions per site. *Sporothrix brunneoviolacea* represents the outgroup.

**Figure S4.** Phylogram from Maximum Likelihood (ML) analysis of *CAL* data for the *Sporothrix stenoceras* & *S. gossypina* complexes. Norwegian and Polish isolates used in this study are in bold. Bootstrap values (if ≥ 75%) for ML and Maximum Parsimony (MP) analyses are presented at the nodes as follows: ML/MP. Bold branches indicate posterior probabilities values ≥ 0.95 obtained from Bayesian Inference (BI) analysis. * Bootstrap values <75%. The tree is drawn to scale (see bar) with branch lengths measured in the number of substitutions per site. *Sporothrix brunneoviolacea* represents the outgroup.
